# Impacts of foodborne inorganic nanoparticles on the gut microbiota-immune axis: potential consequences for host health

**DOI:** 10.1186/s12989-020-00349-z

**Published:** 2020-06-01

**Authors:** Bruno Lamas, Natalia Martins Breyner, Eric Houdeau

**Affiliations:** INRAE Toxalim UMR 1331 (Research Center in Food Toxicology), Team Endocrinology and Toxicology of the Intestinal Barrier, INRAE, Toulouse University, ENVT, INP-Purpan, UPS, 180 Chemin de Tournefeuille, 31027 Toulouse cedex 3, France

**Keywords:** Intestinal microbiota, Gut dysbiosis, Nanoparticles, Silver, Titanium dioxide, Zinc oxide, Silicon dioxide, Gut inflammation, Obesity, Colorectal cancer

## Abstract

**Background:**

In food toxicology, there is growing interest in studying the impacts of foodborne nanoparticles (NPs, originating from food additives, food supplements or food packaging) on the intestinal microbiome due to the important and complex physiological roles of these microbial communities in host health. Biocidal activities, as described over recent years for most inorganic and metal NPs, could favour chronic changes in the composition and/or metabolic activities of commensal bacteria (namely, intestinal dysbiosis) with consequences on immune functions. Reciprocally, direct interactions of NPs with the immune system (e.g., inflammatory responses, adjuvant or immunosuppressive properties) may in turn have effects on the gut microbiota. Many chronic diseases in humans are associated with alterations along the microbiota-immune system axis, such as inflammatory bowel diseases (IBD) (Crohn’s disease and ulcerative colitis), metabolic disorders (e.g., obesity) or colorectal cancer (CRC). This raises the question of whether chronic dietary exposure to inorganic NPs may be viewed as a risk factor facilitating disease onset and/or progression. Deciphering the variety of effects along the microbiota-immune axis may aid the understanding of how daily exposure to inorganic NPs through various foodstuffs may potentially disturb the intricate dialogue between gut commensals and immunity, hence increasing the vulnerability of the host. In animal studies, dose levels and durations of oral treatment are key factors for mimicking exposure conditions to which humans are or may be exposed through the diet on a daily basis, and are needed for hazard identification and risk assessment of foodborne NPs. This review summarizes relevant studies to support the development of predictive toxicological models that account for the gut microbiota-immune axis.

**Conclusions:**

The literature indicates that, in addition to evoking immune dysfunctions in the gut, inorganic NPs exhibit a moderate to extensive impact on intestinal microbiota composition and activity, highlighting a recurrent signature that favours colonization of the intestine by pathobionts at the expense of beneficial bacterial strains, as observed in IBD, CRC and obesity. Considering the long-term exposure via food, the effects of NPs on the gut microbiome should be considered in human health risk assessment, especially when a nanomaterial exhibits antimicrobial properties.

## Background

Nanomaterials are widely used in various industrial manufacturing processes and have applications in everyday consumer products such as foodstuffs, healthcare, clothing, sunscreens and cosmetics. Particles with sizes in the nanoscale range, namely, nanoparticles (NPs; with one dimension between 1 and 100 nm), present unique physical and chemical properties due to their high surface area to volume ratio (e.g., in photocatalysis, mechanical, optical properties), and these properties differ from those of corresponding bulk forms [1–3]. Due to the wide-ranging applications of nanotechnology in everyday products, concerns have been raised regarding the potential health consequences for humans exposed to NPs from different sources and routes (oral, dermal, inhalation). However, the human risk assessment of the oral uptake of NPs is poorly documented compared to that of other routes, although NPs are commonly found in food additives and in food supplements for improving organoleptic properties, shelf life and texture, until used for the nanoencapsulation of specific functional ingredients [4–6].

Among inorganic NPs, titanium dioxide (TiO_2_), silver (Ag) and silicon dioxide (SiO_2_) are commonly used as food colouring or anti-caking agents, while others are added as food supplements, such as zinc oxide (ZnO), given that zinc is an essential trace element. Most of these NPs are also used to develop antimicrobial active food packaging or are incorporated into bio-based materials that come in contact with food to act as oxygen, moisture and carbon dioxide barriers. Oral uptake is thought to be one of the major routes of exposure to NPs for the general population, and studies focused on the fate and effects of foodborne NPs have recently gained considerable attention for the assessment of health risks and for regulatory purposes. Following ingestion, NPs interact with a complex gastrointestinal (GI) environment. The non-absorbed fractions of foodborne mineral NPs (or their ionic forms for soluble compounds, such as Ag and ZnO) are accumulated in the intestinal lumen as a result of daily consumption, and can directly interact with the intestinal microbiota colonizing the gut lumen as well as the mucus layer lining the epithelial surface [7–9]. A portion of the NPs then translocate through the epithelial barrier and are possibly captured by the intestinal immune cells (e.g., macrophages and dendritic cells), before reaching systemic circulation. Notably, the gut microbiota (formerly called the intestinal flora) plays important roles in a number of physiological functions as an indispensable substrate for host health. Indeed, the commensal microbial community not only contributes to digestion of dietary fibres leading to the production of key metabolites for host physiology, but also strongly interacts with epithelial cells to maintain an effective gut barrier separating the body from the external environment [10–12]. Intestinal microorganisms also communicate with local immune cells to shape specific responses by balancing tolerance and effector immune functions to various antigens [10, 13, 14]. Beyond the gut, there is also unique coordination between the intestinal microbiota and liver functions, as well as the brain. Indeed, alteration in the microbiota (namely, dysbiosis), in its ecology (microbial population) and/or metabolic functions (production of bacterial metabolites), has been implicated in various chronic GI and metabolic diseases and even neurodevelopmental disorders [15–21]. For general homeostasis of organisms, immune cells of the gut-associated lymphoid tissue (GALT) shape the microbiota from birth to adulthood in terms of composition, quality and activity and allow these microorganisms to be tolerated by the host for its lifetime. In this context, little attention has been paid to the occurrence of intestinal dysbiosis after daily uptake of nanosized and biocidal particles. Consequences of chronic oral exposure to foodborne NPs on GI functions require scrutiny, taking into account all intestinal compartments that are in close contact with the ingested NPs. This includes direct effects on microbiota composition and/or activity and indirect dysbiosis due to NP-mediated immune system dysfunctions, both leading to potential disruption of intestinal homeostasis, affecting the functions of a number of systemic organs, with long-term effects on health.

Here, we review data from in vivo and in vitro studies on the impacts of Ag, TiO_2_, SiO_2_ and ZnO along the gut microbiota-immune system axis and examine whether these reports are relevant for health risk assessment in humans in terms of dose levels and effects. Silver (referenced as E174 in the EU) is used to colour the surfaces of some products, such as cakes, ice creams, frozen desserts and chocolates, with exposure levels from children to adults ranging from 0.03 to 2.6 μg/kg of body weight (bw)/day (d) [22]. Food-grade TiO_2_ (E171) is a white pigment and brightening agent that is used in large amounts in confectionery items, white sauces and icing [3, 23, 24]. Depending on the exposure scenario, the European Food Safety Authority (EFSA) estimated that the daily exposure levels to E171 range from 0.2 to 1.9 mg/kg bw/d in infants, 0.9 to 10.4 mg/kg bw/d in children, and 0.3 to 6.8 mg/kg bw/d in adults [25]. Food-grade SiO_2_ (E551) is commonly added to powdered food as an anticaking agent, such as in salt, icing sugar, spices, dried milk and dry mixes [26, 27]. The EFSA estimated that daily exposure to E551 ranges between 0.8 and 74.2 mg/kg bw/d in infants, 2.7 and 31.2 mg/kg bw/d in children and 0.9 and 13.2 mg/kg bw/d in adults [28]. In addition, because zinc is important for human health, ZnO-NPs may be found as a nutrient source in supplements and functional foods [29]. To date, the majority of studies evaluating the toxicity of TiO_2_, SiO_2_, Ag and ZnO NPs used nanomodels, i.e., composed of strictly nanosized particles and often coated with different compounds to improve their stability and dispersability. Within the scope of food additives, exhibiting size mix in the nano- and submicron range, with particles as aggregates or agglomerates and batch to batch variation, studies should mainly consider oral exposure to food grade NPs originating from E171, E174 and E551 for human health risk assessment, that remains poorly documented. As most animal studies focused on Ag, TiO_2_, SiO_2_ and ZnO NPs do not address the potential impacts of these materials on the gut microbiota-immune axis, this review will focus on effects susceptible to affect the delicate balance between these microorganisms and their hosts. In addition, we compared the effect of NPs on the gut microbiome to the intestinal dysbiosis characteristics reported in chronic diseases such as colorectal cancer (CRC), obesity and inflammatory bowel diseases (IBD), where microbiota alteration play important pathogenic roles [15–19]. Understanding the impact of NPs on the crosstalk between the microbiota and the immune system will improve the understanding of the potential effects of chronic exposure to foodborne NPs on host physiology. These effects could lead to a favourable environment for the onset and progression of chronic diseases and/or disruption of the gut-liver and gut-brain axes under the influence of bacterial metabolites.

## Main text

### The intestinal microbiota-immune axis in host physiology

The human intestinal microbiota is a complex ecosystem mainly composed of bacteria, as well as archaea, viruses, fungi and protozoa. Colonized at birth, the adult human GI tract harbours 100 trillions of bacteria, including at least several hundred species and more than 7000 strains. A majority of these bacteria belong to the phyla Firmicutes and Bacteroidetes, representing approximately 90% of the microbial population, while other species are members of the phyla Proteobacteria, Verrucomicrobia, Actinobacteria, Fusobacteria and Cyanobacteria [30, 31]. The density of these bacterial populations increases from the proximal to the distal end of the intestine, with ~ 10^2–3^ colony forming units (cfu) per gram in the proximal ileum and jejunum, ~ 10^7–8^ cfu in the distal ileum, and ~ 10^11–12^ cfu in the ascending colon [32]. The biomass of these gut microbes is approximately 1–2 kg, which is similar to the weight of the human brain [33]. The whole genome of intestinal microorganisms (known as the gut microbiome) is 150 times larger than the human genome, providing a diverse range of biochemical and metabolic activities, allowing the microbiota to complement host physiology [31]. Indeed, the gut microbiota and the liver exhibit equal metabolic capacities [34] and due to its beneficial effects for human health, the gut microbiome is now being considered an hidden and indispensable organ of the human body, of which activities need to be integrated as the missing link into host physiology and the development of diseases [35–39].

#### Local and distant roles of the gut microbiome

The intestinal microbiota plays essential roles in GI functions: these microbes i) facilitate the digestion and fermentation of indigestible polysaccharides and produce vitamins, ii) are essential for the development and differentiation of the intestinal epithelium as well as of the GALT, iii) are involved in host immune defence against pathogens in the luminal content, and, finally, iv) contribute greatly to lifelong maintenance of intestinal homeostasis [40–43]. One of the major biological approaches to examine the importance of the gut microbiota for host physiology is the use of germ-free (GF) mice, which are raised in the absence of any microorganisms [44]. GF mice present an underdeveloped intestinal epithelium, which is consistent with the important role of the microbiota in the maintenance of a functional epithelial surface to prevent the passage of harmful intraluminal entities, including foreign antigens, microorganisms and microbial toxins [45–47]. Specifically, GF mice exhibit decreased brush border differentiation [48] and villus thickness [49] due to reduced cell regeneration [50] and increased cell cycle time [51]. Similar to GF mice, GF piglets show aberrant epithelial surface, notably lengthened villi and shortened crypts, and these effects are reversed by intestinal colonization with faecal microbiota or commensal *Escherichia coli* strain [52–55]. Studies in *Drosophila* and mice showed that commensal bacteria promote epithelial development and wound closure through mechanisms that involve reactive oxygen species (ROS) [56, 57]. The intestinal flora also regulates intestinal epithelial cell growth and differentiation indirectly by the production of metabolites such as short-chain fatty acids (SCFAs), and aryl hydrocarbon receptor (AhR) ligands [58–61]. SCFAs (i.e., butyrate, propionate and acetate) are by-products of the fermentation of non-digestible dietary fibres through the action of intestinal bacteria, while AhR ligands are mostly derived from tryptophan metabolism. Moreover, these two metabolite groups are involved in the preservation of intercellular tight junction integrity in the intestinal epithelium, thereby establishing the link between gut microbial activity and the maintenance of effective intestinal barrier function.

Beyond the gut, it is well recognized that the gut microbiome can also modulate the function and/or development of systemic organs, such as the liver and brain, an ongoing dialogue that is enabled by a large variety of bacterial metabolites (SCFAs, polyamines, retinoic acid, AhR ligands). These metabolites can affect distant organs either directly through the bloodstream or indirectly by signalling via nerves or hormones from the gut [20, 62–64]. Both microbiota composition and activity play key roles in such interactions, and dietary alterations may have a significant impact on the gut-liver and gut-brain axes. Consistent with these findings, brain development and social behaviour are impaired in GF mice, and colonization of these mice with a complex microbiota or specific bacterial strains alleviates most of these effects [65–72]. Moreover, GF mice showed an elevated stress response, which was decreased after colonization with wild-type (WT) microbiota or *Bifidobacterium infantis*, a SCFA producer, whereas colonization with the enteropathogenic proteobacteria *E. coli* aggravated the stress response [71]. Intestinal microbiota supplementation with *Lactobacillus reuteri* also restores social behaviours in young mice born to mothers fed a high-fat diet that is known to alter microbiota composition [72]. The social deficit observed in these young mice was not rescued after *Lactobacillus johnsonii* supplementation, indicating that a specific metabolite produced by *L. reuteri* is required [72]. This metabolite remains to be determined, but *L. reuteri* (among other commensals) is able to metabolize the essential amino acid tryptophan to AhR ligands [73, 74] that are known to protect the brain from inflammation [75]. Regarding other bacterial products, the increased blood-brain barrier permeability commonly reported in GF mice was reversed after colonization with the SCFA-producing *Clostridium tyrobutyricum* or *Bacteroides thetaiotaomicron* [69]. SCFAs are also involved in the maturation of microglia, which are responsible for immune defence in the brain [70]. Along the gut-liver axis, AhR ligands such as indole are also able to alleviate liver inflammation in mice [76]. Moreover, SCFA administration in animal models of obesity decreases the accumulation of fat in the liver and improves insulin resistance through mechanisms involving liver glycogenesis and lipogenesis [77]. Again, administration of SCFA-producing bacteria in rats helped prevent non-alcoholic fatty liver disease and lower triglyceride levels [78], reinforcing the idea that the SCFA production level is crucial for liver homeostasis and susceptibility to metabolic diseases.

#### Microbiota and the gut-associated lymphoid tissue

**Fig. 1:**
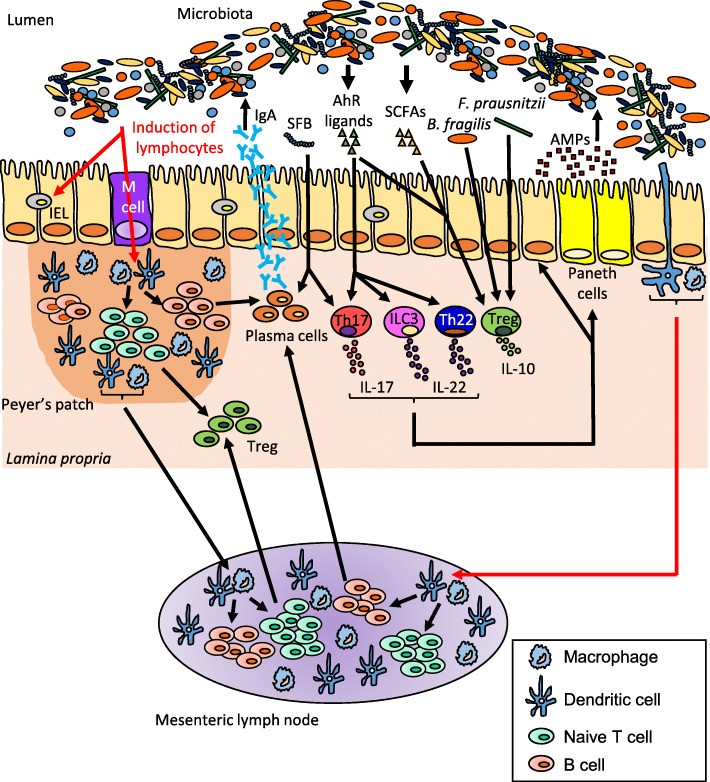
The gut microbiota modulates the intestinal immune response. The gut microbiota influences the development of T cell subsets, intraepithelial lymphocytes (IELs) and are critical for the induction of plasma cells which produce immunoglobulin A (IgA). Dendritic cells (DCs) sample microbial antigens that pass through the epithelial barrier via microfold (M) cells or capture antigens from the lumen directly by extending dendrites between the intestinal epithelial cells. Some of these DCs migrate to the mesenteric lymph nodes and induce naïve T cells differentiation into regulatory T-cell (Treg) by production of transforming growth factor β (TGF-β) and retinoic acid. Segmented filamentous bacteria (SFB) exhibit pro-inflammatory effects by inducing IL-17 and IgA production, whereas *Bacteroides fragilis*, *Faecalibacterium prausnitzii* and short-chain fatty acids (SCFAs) exhibit anti-inflammatory effects via recruitment of Treg that produce the immunosuppressive cytokine IL-10. The intestinal flora also regulates immune response by the production of aryl hydrocarbon receptor (AhR) ligands able to activate AhR, highly expressed on IELs, Th17, Th22, innate lymphoid cells group 3 (ILC3) that produce IL-17 and/or IL-22. These cytokines induce secretion of antimicrobial peptides (AMPs) from Paneth cells and intestinal epithelial cells. AMPs shape the microbiota and are also involved in colonization resistance against pathogens

The intestinal mucosa, encompassing the surface epithelium and the GALT, represents a complex interface participating in the homeostatic relationships between the gut microbiota and the host immune system in response to the environment and diet [79–82]. A balanced dialogue at this interface requires collaboration between mucosal immune cells (macrophages, dendritic cells (DCs), B and T lymphocytes) and epithelial cells producing antimicrobial peptides (e.g., defensins, cathelicidins and histatins) and enzymes such as lysozyme produced by the secretory Paneth cells (Fig. 1). Moreover, GALT also consists of isolated or aggregated lymphoid follicles that form Peyer’s patches (PPs) in the small intestine. PPs exhibit a strong ability to sample luminal antigens and bacteria through microfold (M) cells, a class of antigen-presenting cells and immune sensors of the gut located in the follicle-associated epithelium overlying PP domes (Fig. 1). M cells transport bacteria and macromolecules to the underlying immune cells where they play a critical role in gut-oriented immune responses, triggering the induction of immune tolerance to innocuous antigens derived from food and microbiota or activation of immune defence against pathogens (Fig. 1). In the *lamina propria* (LP), DCs complement these surveillance systems in linking innate immune sensing of the environment to the initiation of adaptive immune responses. By extending transepithelial dendrites into the gut lumen, DCs sample antigens and subsequently migrate to mesenteric lymph nodes (MLNs), where they initiate T-cell responses with an intestinal tropism. Under homeostatic conditions, DCs preferentially promote regulatory T-cell (Treg) responses for oral tolerance [79, 83, 84] while they can also initiate the immunity to pathogens [79, 85] (Fig. 1).

The intestinal microbiota is essential for GALT maturation and development, as proven by GF mice exhibiting highly immature immune system. The intestine of these mice exhibit small MLNs and PPs and decreased numbers of immune cells, such as immunoglobulin A (IgA)-producing plasma cells and T lymphocytes, for example, LP CD4^+^ T cells and intraepithelial αβ CD8^+^ T cells, leading to a reduced capacity to combat pathogens [40, 86]. All these immune alterations are microbiota dependent and can be reversed by faecal microbiota colonization [87]. Notably, recent studies highlighted the existence of anti- or pro-inflammatory microorganisms in the gut. In mice, segmented filamentous bacteria (SFB) direct the accumulation of pro-inflammatory T helper 17 (Th17) cells in the LP [88, 89] (Fig. 1). This finding is in contrast with the results for other bacteria, such as *Bacteroides fragilis* [90, 91] and *Faecalibacterium prausnitzii* [92–94], which exhibit anti-inflammatory activity via metabolite production and/or the recruitment of Treg-producing interleukin (IL)-10, a potent immunosuppressive cytokine (Fig. 1). Indeed, most metabolites derived from commensal bacteria, such as SCFAs and AhR ligands, may regulate immune cell functions via indirect and direct mechanisms [10, 74, 95] (Fig. 1). In the intestine, butyrate is a key regulator of host energy, and the butyrate level in the lumen is correlated with the abundance of *Lactobacillus*, *Allobacterium*, *Bifidobacterium*, *Dorea* and *Blautia*. Many cells are affected by SCFAs, such as intestinal epithelial cells (IECs), DCs and T cells, leading to modulation of different aspects of cell development, survival and function and to modulation of enzymes and transcription factors [96, 97]. Gurav and colleagues demonstrated that SCFAs modulate T cell activation and effector response by inducing a tolerogenic profile [98]. This study also showed that DCs stimulated with SCFAs in vitro exhibited increased expression of indoleamine 2,3-dioxygenase 1 [98], the role of which in inflammation is to attenuate the immune response through an immunosuppressive effect on T cell proliferation and activation. On the other hand, AhR ligands produced by the gut microbiota induce AhR activation, with endogenous downstream functions on the cell cycle, immune response, and cell differentiation [99–101] (Fig. 1). IECs have a role in the regulation of AhR ligands availability to GALT, including macrophages, DCs, innate lymphoid cells (ILCs), Th17/Th22 cells and intraepithelial lymphocytes (IELs). All of these immune cell types express AhR, activation of which by microbiota-derived appropriate ligands being pivotal in the regulation of mucosal immune responses in the gut [99].

### Interactions of inorganic NPs with the gut microbiome

Despite several studies highlighting the deleterious effects of nanomaterials in soil [102] or plant [103] microbial communities, the impact of inorganic NPs on bacteria that colonize the gut remain poorly documented. Tables 1 and 2 provides a detailed description of data on NPs that exhibit biocidal effects on intestinal bacteria, mainly metal NPs such as Ag, ZnO and TiO_2_, with additional information for SiO_2_. Given the very limited information on nanostructured food additives (namely, E174, E171, E551 in EU for food-grade formulations of nano-Ag, TiO_2_ and SiO_2_, respectively), it should be noted that this review mostly provides data obtained with NP models. The in vivo studies are categorized according to the duration of treatment and dose levels (Table 1), followed by classification of in vitro data on the human microbiota (Table 2), to provide information relevant for risk assessment of NP exposure through food.

**Table 1:** Studies using animal models to determine the impact of NPs on the gut microbiota

	Ag-NP		TiO_2_-NP		SiO_2_-NP	ZnO-NP	
Duration (days)	≤14	≥28	7	≥28	7	≤14	≥28
Actinobacteria				↗[104]			
*Bifidobacterium (g)*		↙[105]					
*Corynebacterium (g)*	↙[106]						
*Rhodococcus (g)*				↙[104]			
Bacteroidetes	↗[107]	↗[105] or ↙[108]^♦^	↙[107]^♦^	↙[104]	↙[107]^♦^	↙[109]	↗[110]
*Bacteroidaceae (f)*		↙[108]^♦^					↗[110]
*Bacteroides (g)*		↗[105] or ↙[108]^♦^	↙[107]^♦^		↙[107]^♦^		↗[110]
*Bacteroides uniformis*	↙[106]						
*Odoribacteraceae (f)*		↙[108]^♦^					
*Prevotellaceae (f)*						↙[109]	
*Prevotella (g)*	↗[107]			↙[104]	↗[107]^♦^	↙[109]	
*Rikenellaceae (f)*		↗[108]^♦^					↙[110]
*Alistipes (g)*	↗[107]				↗[107]^♦^		↙[110]
*S24–7 (f)*		↙[108]^♦^				↙[109]	
*Barnesiella (g)*				↙[104]			
Firmicutes	↙[107]	↙[105] or ↗[108]^♦^		↙[104]	↗[107]^♦^	↗colon, ↙ileum [109]	↙[110]
*Bacillaceae (f)*						↗[109]	
*Bacillus (g)*						↗[109]	
*Christensenellaceae (f)*	↙[106]						
*Erysipelotrichaceae (f)*				↙[104]			
*Turicibacter (g)*				↙[104]			↗[111]
*Lachnospiraceae (f)*	↙[107]	↗[108]^♦^			↗[107]^♦^		
*Blautia (g)*		↗[108]^♦^					
*Coprococcus (g)*		↗[108]^♦^					
*Coprococcus eutatus*	↙[106]						
*Lactobacillaceae (f)*		↗[108]^♦^				↗colon, ↙ileum [109]	↙[110, 111]
*Lactobacillus (g)*	↙[107]	↙[105] or ↗[108]^♦^		↙[104]	↙[107]^♦^	↗colon, ↙ileum [109]	↙[110, 111]
*Peptococcaceae (f)*	↙[106]						
*Ruminococcaceae (f)*					↗[107]^♦^		
*Oscillospira (g)*	↙[106]					↙[109]	
*Streptococcaceae (f)*						↗[109]	
*Streptococcus (g)*						↗[109]	
*Clostridium (g)*	↙[106]						↗[111]
*Dehalobacterium (g)*	↙[106]						
*Oscillibacter (g)*					↗[107]^♦^		
*SMB53 (g)*							↗[111]
Proteobacteria				↗[104]	↗[107]^♦^	↗[109]	↙[111]
*Enterobacteriaceae (f)*		↗[105]					
*Escherichia/Shigella (g)*				↗[104]			
*Halomonadaceae (f)*						↗[109]	
*Halomonas (g)*						↗[109]	
*Aggregatibacter pneumotropica*	↙[106]						

^♦^ Studies using NPs at human relevant doses

**Table 2:** In vitro studies exploring the impact of NPs on human microbiota

	Ag-NP	TiO_2_-NP
Duration (days)	2	2
Bacteroidetes		
*Bacteroides ovatus*	↙[112]	↙[113]
Firmicutes		
*Acidaminococcus intestini*		↗[113]
*Clostridium cocleatum*		↗[113]
*Eubacterium rectale*	↙[112]	↗[113]
*Eubacterium ventriosum*		↗[113]
*Faecalibacterium prausnitzii*	↙[112]	
*Roseburia faecalis*	↙[112]	
*Roseburia intestinalis*	↙[112]	
*Ruminococcus torques*	↙[112]	
Proteobacteria		
*Escherichia coli*	↗[112]	
*Raoultella (sp)*	↗[112]	

#### Impacts of nano-silver and titanium dioxide

Regarding Ag-NPs, several studies were first performed in rats or mice using high concentrations (up to 36 mg/kg bw/d) compared to human dietary levels (0.03 to 0.65 μg/kg bw/d [22]) and with different methods of administration (gavage, addition to drinking water or incorporation into food pellets) and/or exposure times (between 7 and 90 days). Contradictory results were reported, with some studies showing no impact on microbiota ecology and activity and, others revealing profound alterations. Indeed, two studies in rats and mice (9 and 10 mg/kg bw/d, respectively) showed non-significant alterations in the caecal microbiota composition following 28 days of oral exposure to Ag-NPs (stabilized with polyvinylpyrolidone or citrate), regardless of particle size (14, 20 and 110 nm) [114, 115]. In contrast, rats chronically exposed for 13 weeks to Ag-NPs (10, 75 and 110 nm at 9, 18 and 36 mg/kg bw/d) showed a shift in their ileal microbial populations towards increased proportions of Bacteroidetes and pathogenic gram-negative bacteria, and decreased proportions of Firmicutes, *Lactobacillus* and *Bifidobacterium* [105]. These effects on *Lactobacillus* and Bacteroidetes appeared to be more prominent in males than in females, whereas the increased proportion of Enterobacteria was higher in female rats [105]. In addition to the dose of exposure, authors considered NP size as an influencing factor on host gene expression, but the heterogeneity of the results does not allow to fully conclude on a size and/or dose effect of Ag-NPs [105]. The shift in the Firmicutes/Bacteroidetes (F/B) ratio has been confirmed in the faecal microbiota of rats and mice orally given Ag-NPs at 2.5 or 3.6 mg/kg bw/d for 7 days or 2 weeks, respectively [106, 107]. Notably, these authors concluded that microbiota alterations depend on the shapes of Ag-NPs [106], suggesting that NPs structure (which affects the surface-to-volume ratio) could determine the reactivity with gut bacteria. Finally, a shift in the F/B ratio in the faecal microbiota has been reported in mice orally exposed for 28 days to food supplemented with relevant doses of Ag-NPs for human intake [108]. The authors noted that these gut microbiota alterations did not occur when mice were fed aged food pellets due to sulfidation of Ag-NPs in contact with the sulfur-containing food matrix [108]. Because Ag sulfidation limits the release of the toxic Ag^+^ ions responsible for antimicrobial effects, one may hypothesize that this ageing mechanism could contribute to the aforementioned discrepancies in rodent studies. Some differences in terms of gut microbiota alterations might also be due to differences in NP doses, durations of treatment, microbiota sampling sites along the rodent intestine (i.e., small bowel or more distal regions in the caecum or colon), and the methods and analysis techniques used to determine bacterial composition. Regarding the sampling site, the faecal samples from the distal colon should remain the predominant material for microbial community analysis in toxicity testings with NPs, because this region is characterized by a low transit time making reservoir for NP accumulation after chronic exposure, as reported in rats daily treated with E171 [116], with expected stable impacts on the microbiome.

Although the impacts of Ag-NP ingestion on the rodent microbiota are well documented, their effects on the human microbiota remain to be fully characterized. Only one study has determined, in vitro, the short-term impacts of Ag-NPs on a defined human bacterial community called microbial ecosystem therapeutic-1 (MET-1, consisting of 33 bacterial strains, established from stool obtained from a healthy donor) [112]. The authors observed a reduction in culture-generated gas production and changes in fatty acid methyl ester profiles after 48 h of exposure to several concentrations of Ag-NPs (0–200 mg/L) [112]. Microbiota sequencing confirmed alterations in bacterial composition, characterized by decreased abundances of *Bacteroides ovatus*, *F. prausnitzii*, *Roseburia faecalis*, *Roseburia intestinalis*, *Eubacterium rectale* and *Ruminococcus torques*, and increased proportions of *Raoultella* sp. and of *E. coli*, i.e., a negative shift in the microbial community that favours the growth of pathogenic bacteria [112].

The same bacterial community, MET-1, has also been used in a custom colon reactor to determine the impact on the human microbiota of TiO_2_-NPs and different food-grade TiO_2_ formulations (the E171 food additive in EU) [113]. A minor effect on bacterial ecology, restricted to a decrease in *B. ovatus* in favour of *Clostridium cocleatum*, was reported after 48 h of TiO_2_ treatment [113]. However, using long exposure times in vitro (5 days) at environmentally relevant concentrations, significant changes in bacterial metabolites were observed in the human colon microbiome, including in SCFA production [117]. To date, whether the effect of TiO_2_ (on phyla, strains and/or metabolic activity) could occur in vivo at dietary levels for humans has remained poorly studied. Nevertheless, oral bioavailability studies in rodents and humans clearly showed very limited systemic absorption of TiO_2_ (0.1 to 0.6% of the initial dose, respectively) [118, 119]. This finding indicates that at least 99% of the ingested TiO_2_ matter accumulates in the lumen of the gut with the commensals in permanent contact with the particles, especially due to repeated exposure, with the potential for alterations in the growth profiles of bacteria as shown in vitro for E171 [8]. Mice exposed for one week to TiO_2_-NPs at a relevant dose for humans (2.5 mg/kg bw/d) did not reveal any changes in the faecal microbiota composition [107]. However, an increased proportion of potentially harmful Actinobacteria and Proteobacteria and a decrease in the abundance of beneficial Firmicutes and Bacteroidetes were observed in the same region after 28 days of oral treatment, but at higher dosage (100 mg/kg bw/d) [104]. Currently, due to the absence of specific tests for assessing NP-related changes in the composition and activity of the gut microbiota, it is important to elucidate the potential induction of dysbiosis by TiO_2_-NPs to perform these examinations at appropriate doses and over long periods for relevant dietary exposure models.

#### Impacts of zinc oxide and silicon dioxide

Zinc oxide NPs exhibit potent antimicrobial activity on various non-intestinal microorganisms. ZnO is currently listed as a generally recognized as safe (GRAS) material by the Food and Drug Administration and is commonly used as food supplement or in food packaging. Due to a widespread use to enhance the bioavailability of zinc in the body, these metal particles are good candidates for NP-induced intestinal dysbiosis, but this aspect has been poorly explored to date. Most of the available studies have been conducted using the microbiota from piglets and hens due to efficient functioning of ZnO to promote growth and relieve diarrhoea in livestock animals [120]. The ingestion of ZnO-NPs at 600 mg/kg for 14 days increased the bacterial richness and diversity in the ileum of piglets, while these parameters decreased in both caecum and colon [109]. Nevertheless, the authors observed different effects on microbiota composition according to the collection sites along the GI tract. An increased abundance of *Streptococcus* concomitant with a decreased proportion of *Lactobacillus* was observed in the ileum, while the abundance of *Lactobacillus* increased and the abundance of *Oscillospira* and *Prevotella* decreased in the colon [109]. In hens, gene-sequencing analysis of the 16S rRNA of the ileal digesta microbiota showed that the richness of the bacterial community decreased in a dose-dependent manner (25, 50 and 100 mg/kg for 9 weeks), with increased populations of Bacteroidetes, Fusobacteria and Bacilli and decreased populations of Proteobacteria and *Lactobacillus* [111]. Moreover, blood metabolite analysis clearly indicated a positive correlation between the richness of the microbiota and choline, lactate and methionine metabolism, suggesting an impact of ZnO-NPs on bacterial metabolic activity [111]*.* These observations are consistent with those of another study that showed decreased abundance of the SCFA-producing *Lactobacillus* in the caecum of broiler chickens fed ZnO-NPs at 5 mg/kg of feed for 42 days [110]. One study focused on the human faecal microbiota isolated from a healthy donor confirmed the impacts of ZnO-NPs on the metabolic activity of the gut microbiota, impairing the production capacity of SCFAs as well as extracellular polymeric substances (i.e., polysaccharides, proteins, lipids, and extracellular DNA secreted by bacteria in protective biofilms) [117]. Overall, these studies showed that oral ingestion of ZnO-NPs can lead to changes in both the composition and metabolic activity of the intestinal microbiota, the main feature being decreased abundance of the genus *Lactobacillus* regardless of the animal model. Finally, with regard to non-metal NPs without well-known biocidal properties, one study in mice exposed to SiO_2_-NPs for one week at relevant dose for humans (2.5 mg/kg bw/d) reported enrichment and increased diversity of microbial species, with increased populations of Firmicutes and Proteobacteria and decreased proportion of Bacteroidetes and *Lactobacillus* [107]. Such an unexpected effect should be taken into consideration for risk assessment in the context of the low absorption rate in the human GI tract for precipitated or fumed (amorphous) silicate (E551) [28], leading to gut lumen accumulation.

### Microbiota-immune system dysfunction in chronic diseases: could inorganic NPs favour host vulnerability?

#### NP-related GALT dysfunctions as possible inducers of disease development

Despite several in vitro studies highlighting the variety of immunotoxic effects of TiO_2_-NPs, SiO_2_-NPs, Ag-NPs and ZnO-NPs (Table 3) on bone marrow-derived cells and systemic and pulmonary immune cells [121–151], the potential impact of these NPs on GALT functions and consequences for the host remain poorly documented. From a risk perspective, current challenges include the determination of whether NP-related dysbiosis and immune dysfunction may increase the susceptibility to chronic diseases in humans where disruption of the microbiota-GALT crosstalk is central to pathogenesis, such as in CRC, obesity and IBD [13, 152, 153]. IBD, namely Crohn’s disease and ulcerative colitis, are chronic relapsing disorders of multifactorial aetiology characterized by intestinal dysbiosis and severe mucosal inflammation with epithelial injuries. In addition, gut dysbiosis and low-grade inflammation of the intestinal mucosa are often viewed as contributors to CRC and obesity [13, 152, 153]. Notably, micro- and nano-particles have been found in colon biopsies of patients with IBD and CRC, whereas the absence of these particles is consistently reported in the colon of healthy subjects [154]. Again, blood titanium (Ti) levels are high in IBD patients [155], and TiO_2_ particles of dietary origin were shown to be accumulated in PPs of IBD patients [156], including infants [157]. Such localization is consistent with data from studies in rats and mice that commonly report bioaccumulation of Ti, Ag or silicon (Si) in the ileum (including PPs) and the colon as a result of repeated oral treatment with TiO_2_-NPs (or E171) [107, 116, 158, 159], nano-Ag [160] or SiO_2_-NPs [107], respectively. In the colon of rats chronically exposed for 100 days to food-grade TiO_2_ (E171) at a relevant dose for humans (i.e., 10 mg/kg bw/d), a micro-inflammatory state of the mucosa has been demonstrated [116]. Using a ten-fold higher dosage, another study in mice exposed to TiO_2_ particle models (nano- and microsized) found increased production of Th1 pro-inflammatory cytokines (tumour necrosis factor alpha (TNF-α), interferon-gamma (IFN-γ), IL-12) and of the Th2 cytokine IL-4 in the mucosa of the small intestine after 10 days of exposure [161]. Inflammation associated with other immune system disturbances has also been observed following the ingestion of SiO_2_-NPs [107, 162] and Ag-NPs [107, 163], including impairment of oral tolerance mechanisms to food proteins and bacterial antigens present in the gut lumen [162, 163]. Furthermore, IBD-like symptoms, including intestinal upregulation of pro-inflammatory cytokines, were notably induced in mice treated with Ag-NPs at 2.5 mg/kg bw/d for 7 days [107]. Again, oral administration of TiO_2_-NPs worsened existing gut inflammation in a mouse model of acute colitis through activation of the inflammasome [155]. As an exception, the intestinal expression of pro-inflammatory genes was decreased in piglets exposed orally to ZnO-NPs for 2 weeks [109], which is consistent with ZnO-NPs exhibiting markedly dose-dependent effects on the remission of experimental colitis in mice [164].

**Table 3:** In vitro studies on immunological properties of NPs

NPs	cell response	cell frequence/activity	Potential effect
TiO2 (6-48 h)	**↗** oxidative stress (ROS) [121–125]	**↗** DC, μɸ, lymphocytes [121, 122, 126]	Innate inflammatory response [122, 126–128]
	**↗** expression of TLR (TLR3, TLR4, TLR7 and TLR10) [126, 128]	**↗** naïve T cells [122]	Cytotoxicity and inflammation [122, 123, 125, 129]
	**↗** pro-inflammatory cytokines (IL-6, TNF-α, IL1-β) [121–123, 127, 130–132]	**↗** mature DC [122, 131, 132]	Adjuvant [121, 130–132]
	**↗** chemokines secretion (IL-8 and CXCL1) **↙** activity (IL-8) [122, 133]	**↗** mast cell activation [134]	Allergic response [134, 135]
	**↗** co-stimulatory molecules (CD80 and CD86) [122, 123, 131, 132]	**↙** neutrophil and μɸ mobility [133]	Tissue damage [124, 133]
	**↗** MPO, MMP-9 and NET [124, 136]	**↗** neutrophil activity [124, 136]	NETosis: inflammation, necrosis and apoptosis [124]
	**↗** β-hexosaminidase release [134]		
	inflammasome activation [123]		
	activation of NFkB pathway [128, 131]		
SiO2 (6-48 h)	**↗** apoptose [123, 125, 137, 138]	**↙** DC and lymphocytes [123, 137, 138]	Imbalance of immune response [125, 126, 137–140]
	**↗** pro inflammatory cytokines (IL1-β, IL-2, TNF-α) [121, 123, 126, 138, 139, 141]	**↗** DC maturation [123, 139]	Immunogenic or adjuvant potential [121, 123, 126, 140, 141]
	**↗** CD80, CD86 and MHCII [123, 138, 139]	**↗** M1 μɸ polarization [126, 140]	Inflammation [121, 123, 125, 126, 137–141]
	**↙** TLR9 expression [126]	**↗** neutrophil activity [142]	**↗** Susceptibility IBD [123, 126, 140], autoimmune diseases [139, 140]
	**↗** oxidative stress (ROS) [125]	**↗** cross-presentation [141]	NETosis: inflammation, necrosis and apoptosis [142]
	inflammasome activation [123]		Cytotoxic effect [121, 123, 125, 137, 138, 140]
	NFkB activation [138]		Susceptibility to infection [126]
	**↗** DNA release – NET [142]		Allergic response [135]
ZnO (6-48 h)	**↗** pro inflammatory cytokines (IL-1β, TNF-α, IL-6, IFN-ɣ) [125, 130, 143, 144]	**↗** DC activity [130, 145]	Cytotoxicity and inflammation [125, 129, 130, 134, 143, 146]
	**↗** chemokines secretion (IL-8, CXCL9) [130, 144]	**↙** Lymphocytes [146]	Imbalance of immune response [125, 129, 130, 143, 144]
	**↗** oxidative stress (ROS) [125, 145]	**↗** neutrophil functions [136]	Chronic pathologies [143]
	induces neo-synthesis of polypeptides [144]	**↙** mast cell activation [134]	Allergenic response [134, 135]
	**↙** or **↗** apoptose [125, 129, 130, 144]	**↗** eosinophils [144]	Protective effect [134, 144]
	**↗** DNA damage [125, 129, 146]		Genomic instability [146]
	**↙** β-hexosaminidase and histamine release [134]		Cell cycle imbalanced [125]
Ag (6-48 h)	**↗** oxidative stress (ROS) [147–149]	**↙** μɸ, lymphocyte [147–149]	Apoptosis and cytotoxicity [147–149]
	**↗** DNA damage [148]	**↗** mast cell activation [135, 150, 151]	Inflammation/imbalance of immune response [147–149]
	**↗** pro inflammatory cytokines (IL-1β, TNF-α) [147, 149]		Allergic response [135, 150, 151]
	inflammasome activation [147]		
	**↗** β-hexosaminidase release [135, 150, 151]		

*CD* Cluster of differentiation, *CXCL1* chemokine ligand 1, *DC* dendritic cell, *IBD* Inflammatory bowel disease, *Ig* immunoglobulin, *IL* interleukin, *μɸ* macrophage, *MHCII* major histocompatibility complex II, *MMP-9* matrix metalloproteinase 9, *MPO* myeloperoxidase, *NET* Neutrophil extracellular trap, *NFkB* nuclear factor-kappa B, *ROS* reactive oxygen species, *TLR* Toll-like receptor

**Fig. 2:**
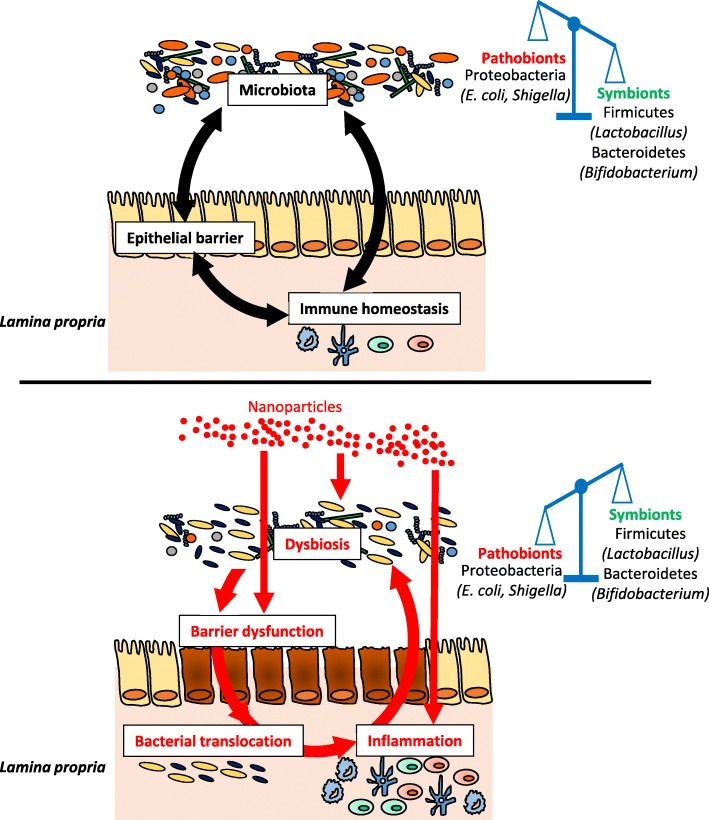
Potential impact of NP ingestion on the crosstalk between the microbiota and the immune system. After ingestion, NPs interact with the gastrointestinal environment and can alter the gut microbiota, characterized by an alteration of the F/B ratio, a depletion of *Lactobacillus* strains and an increase in the abundance of Proteobacteria. NPs exhibit also deleterious effects on the epithelial barrier and the intestinal immune response, which can amplifies the dysbiosis in a vicious circle favouring intestinal inflammation in susceptible individuals

Taken together, these data highlight the considerable impact of TiO_2_-NPs, Ag-NPs and SiO_2_-NPs on the modulation of the immune response in vivo (Table 4) [105, 116, 161–163, 165–170], which may in turn modulate the microbiota. Indeed, recent studies in mice have shown a number of immune deficiencies evoking intestinal dysbiosis in ways that predispose to disease. For example, mice lacking the caspase recruitment domain 9 (*Card9*^−/−^, a key adapter protein for innate immunity against a wide range of microorganisms) developed colitis in a microbiota-dependent manner [74]. Remarkably, this colitis phenotype was transmissible to WT mice via transfer of the *Card9*^−/−^ microbiota and was rescued after inoculation with *Lactobacillus* strains [74]. Similarly, immune-driven dysbiosis was demonstrated in mice lacking Toll-like receptor 5 (*Tlr5*^−/−^), which detects bacterial flagellin. The mice exhibit an altered microbiota associated with metabolic disorders characterized by insulin resistance, hyperlipidaemia and increased fat deposition, and all these markers were also observed in WT mice colonized with the gut microbiota of Tlr5-deficient mice [171]. By describing the key role of the local immune system in controlling the composition and activity of the microbiota, these data support the idea that the strong interactions between NPs and GALT can also shape the microbiota of exposed individuals (Fig. 2).

**Table 4:** *In vivo* studies on immunological properties of NPs

NPs	Time	Cell response	Cell frequency/activity	Dose range	Potential effect	Ref
TiO_2_	1 -14 d	↗ IFN-ɣ and IL-17 in spleen	↗ Th17 and Th1 in spleen	**10** mg/kg bw/d	inflammation	[116]
			↗ DC frequency in PP		imbalance immune response	
			↙ Treg and T helper cells in PP		immunossuppression	
		↗ inflammatory cytokines in ileum (IL-12, IL-4, IL-23, TNF-α, IFN-γ)	↗ T cells CD4^+^ in small intestin	100 mg/kg bw/d	inflammation	[161]
				5 g/kg bw (single oral gavage)	no toxic effects	[165]
	> 28 d	↗ TNF-α, IL-10 and IL-8 in colon	↙ Treg and T helper cells in PP	**10** mg/kg bw/d	risk of preneoplasic lesions in colon	[116]
		↗ IL-6, TNF-α, IL1-β in ovary (expression and production)	↙ white cells and lymphocytes in blood	**2.5; 5; 10** mg/kg bw/d	inflammation and follicule atresia	[166]
					fertility reduction	
		↗ glucose in blood of young rats	↗ mast cells activation in stomach	**10**, 50, 200 mg/kg bw/d	alteration of gastrointestinal functions	[167]
					Heart, Liver and Kidney injuries	
SiO_2_	7 -14 d	↙ inflammatory cytokines in blood (IL1-β, TNF-α, IL-12p70, IL-6 and IFN-ɣ)	↙ Natural Killer acitivity in spleen	750 mg/kg bw/d	immunossuppression	[168]
			↙ T cells and B cells proliferation in spleen		dysregulation of immune response	
			↙ Lymphocytes population in blood			
		↗ IgG and IgE in blood	↗ splenocyte proliferation	**0.1**; 1; 10 mg/mouse/d	imbalance immune response (dose dependent)	[162]
		↗ inflammatory cytokines in spleen (INF-ɣ, IL-4, IL-5 and IL-17)			risk of food allergy development	
	≥ 28 d		slight ↗ lymphocytes in blood	2.5 mg/mouse/d	no effect	[169]
Ag	7-14 d	↗ TGF-β in blood	↗ B and Natural Killer cells in blood ↗ T CD8^+^ population in blood	1 mg/kg bw/d	imbalance immune response	[170]
		↗ IgG in blood, ↗ TGF-β and IFN-ɣ in spleen	↗ splenocyte proliferation	0.01; 0.1; 1; 10 mg/kg bw/d	imbalance immune response	[163]
	≥ 28 d	↗ inflammatory cytokines in blood (IL1-β, IL-6, IL-4, IL-10, IL-12 and TGF-β)	↗ T CD8^+^ population in blood	0.25; 0.50; 1 mg/kg bw/d	inflammation	[170]
		↗ IgE in blood	↗ B cell in blood		risk of food allergy development	
		↙ gene expression in ileum (MUC3, TLR2, TLR4, FOXP3, IL-10, TGF-β)		9 mg/kg bw/d	immunossuppression	[105]
					sexual dimorphism response	

The relevant doses for humans are indicated in bold. *DC* dendritic cell, *FOXP3* forkhead box P3, *GPR43* G-protein coupled receptor 43, *IFN-γ* interferon-gamma, *Ig* immunoglobulin, *IL* interleukin, *MUC3* mucin 3, *NOD2* nucleotide-binding oligomerization domain 2, *TGF-β* transforming growth factor beta, *Th* T helper, *TLR* Toll-like receptor, *TNF-α* tumor necrosis factor alpha, *Treg* regulatory T-cell

#### The NP-induced gut microbiota signature resembles that of dysbiosis-associated human diseases

Despite some contradictory studies, the majority of observations (Table 1) [104–113] reveals a microbiota signature for nano-Ag, TiO_2_, ZnO and SiO_2_ characterized by alteration of the F/B ratio together with depletion of *Lactobacillus* and enrichment of Proteobacteria. The F/B ratio is often considered an informative parameter for the general state of the intestinal microbiota. Alteration of the F/B ratio has been observed in diseases associated with dysbiosis [172–175], and helps to predict the decrease in the relative abundance of SCFAs [176, 177]. Furthermore, *Lactobacillus* confers a health benefit on the host, notably via SCFAs and AhR ligand production [73, 74, 99, 178–180], while pathogenic Proteobacteria (*E. coli*, *Shigella*, *Listeria*, etc.,) are often overrepresented in several intestinal and extra-intestinal diseases with an inflammatory phenotype [181, 182]. Altogether, these data emphasize a negative shift in the microbial community in response to NP exposure, favouring the growth of pathogenic bacteria at the expense of beneficial strains such as *Lactobacillus* (Fig. 2). Importantly, these reported effects were very similar to those observed in patients suffering from IBD, CRC or chronic metabolic disorders such as obesity (Table 5) [17, 92, 173, 183–222].

**Table 5:** Microbiota alteration observed in IBD, CRC and obesity compared to those induced after NP ingestion

	IBD	CRC	Obesity	NPs effects on microbiota
Actinobacteria	↗[183–185]	↗[186]	↗[187]	↗TiO_2_ [104]
*Acidimicrobidae ellin* 7143	↗[184]			
*Actinobacterium* GWS-BW-H99	↗[184]			
*Actinomycinaeae (o)*	↗[184]			
*Actinomyces (g)*	↗[188]			
*Actinomyces oxydans*	↗[184]			
*Bifidobacteriales (o)*		↗[186]		
*Bifidobacterium (g)*	↙[189]	↙[190, 191]	↙[192, 193]	↙Ag [105]
*Bifidobacterium adolescentis*	↙[194, 195]			
*Corynebacteriaceae (f)*	↗[184]		↗[196]	
*Nocardioides* NS/27	↗[184]			
Bacteroidetes	↙[183, 184, 188]	↙[17, 197]	↙[173, 187, 198–200]	↙Ag, TiO_2_, SiO_2_, ZnO [104, 107–109]; ↗Ag, ZnO [105, 107, 110]
*Bacteroides (g)*	↙[183, 194]	↙[17, 186, 197, 201]	↗[202, 203]	↙Ag, TiO_2_, SiO_2_ [107, 108]; ↗Ag, ZnO [105, 110]
*Bacteroides ovatus*	↙[204, 205]		↙[206]	↙Ag, TiO_2_ [112, 113]
*Bacteroides uniformis*	↙[183]	↙[186]	↙[206]	↙Ag [106]
*Prevotellaceae (f)*			↗[196, 207]	↙ZnO [109]
*Prevotella*	↙[183]	↙[201]	↗[207, 208]	↙TiO_2_, ZnO [104, 109]; ↗Ag, SiO_2_ [107]
*Rikenellaceae (f)*	↙[209]		↙[200, 207]	↙ZnO [110]; ↗Ag [108]
*Alistipes (g)*	↙[184, 185, 188]	↗[210]	↙[193, 206]	↙ZnO [110]; ↗Ag, SiO_2_ [107]
Firmicutes	↙[92, 184, 211, 212]	↙[197]	↗[173, 199, 200]	↙Ag, TiO_2_, ZnO [104, 105, 107, 109, 110]; ↗Ag, SiO_2_, ZnO [107–109]
*Erysipelotrichaceae (f)*	↙[194]	↗[17, 190]	↗[196]	↙TiO_2_ [104]
*Turicibacter (g)*	↙[183]			↙TiO_2_ [104]
*Lachnospiraceae (f)*	↙[194, 209]	↙[197, 201]	↙[207]	↙Ag [107]; ↗Ag, SiO_2_ [104, 107]
*Blautia (g)*	↙[209]	↙[17]	↙[207]	↗Ag [108]
*Blautia faecis*	↙[183]			
*Coprococcus (g)*	↙[209]		↙[193, 207]	↗Ag [108]
*Roseburia (g)*	↙[185, 209]	↙[186, 210]	↙[207]	
*Roseburia intestinalis*	↙[183, 194]		↗[206, 213]	↙Ag [112]
*Roseburia inulinivorans*	↙[183]			
*Clostridium XIVa* and *IV* groups	↙[92, 184]		↗[206]	
*Clostridium lavalense*	↙[183]			
*Dialister invisus*	↙[195]			
*Enterococcus (g)*		↗[186]		
*Eubacterium (g)*		↙[186]	↙[207]	
*Eubacterium rectale*	↙[194, 211]		↗[213]	↙Ag [112]; ↗TiO_2_ [113]
*Eubacterium ventriosum*	↙[183]		↗[198, 213]	↗TiO_2_ [113]
*Faecalibacterium (g)*	↙[185, 209]	↙[17, 190, 197, 210]	↙[193]	
*Faecalibacterium prausnitzii*	↙[92, 183, 194, 211]	↙[191, 210]	↙[206, 214]	↙Ag [112]
*Lactobacillus (sp)*	↙[183]	↙[191, 215]	↙[193, 216]	↙Ag, TiO_2_, SiO_2_, ZnO [104, 105, 107, 109–111]; ↗Ag, ZnO [108, 109]
*Oscillospira (g)*			↙[207, 213]	↙Ag, ZnO [106, 109]
*Ruminococcus gnavus*	↗[195, 209, 217]			
*Ruminococcus torques*	↙[183, 194]			↙Ag [112]
*Streptococcus (g)*		↗[186, 218]		↗ZnO [109]
Proteobacteria	↗[184, 185]	↗[186]	↗[200, 206, 216]	↗TiO_2_, SiO_2_, ZnO [104, 107, 109]; ↙ZnO [111]
*Enterobacteriaceae (f)*	↗[209, 219]	↗[191, 218]	↗[207]	↗Ag [105]
*Escherichia coli*	↗[194, 211, 220–222]	↗[186, 210]		↗ Ag, TiO_2_ [104, 112]
*Shigella (sp)*	↗[185, 211]	↗[186, 210]		↗ TiO_2_ [104]
*Sutterella (g)*			↗[216]	
*Listeria (sp)*	↗[211]			

A deficiency in AhR ligand production by the microbiota was reported in IBD and obese patients compared to healthy subjects [74, 178]. Some commensal bacteria are able to metabolize the essential amino acid tryptophan in AhR ligands such as indole, indole-3-acetic acid, tryptamine, and indole-3-aldehyde. This ability is mainly exhibited by *Lactobacillus* [73, 74, 99, 178], the abundance of which is decreased in the microbiota of IBD and obese patients [183, 193, 216]. Recent studies in mice demonstrated that the reduced capacity of the microbiota to produce AhR ligands is involved in the pathogenesis of IBD and obesity through a mechanism that involves decreased IL-22 production by intestinal immune cells [74, 178]. In the intestine, IL-22 is involved in mucosal wound healing [223] and production of antimicrobial peptides (AMPs) by IECs, such as regenerating islet-derived 3 gamma (Reg3γ) and Reg3β [224, 225], which can modulate the microbiota composition. Interestingly, defective intestinal production of IL-22 was observed in mice fed a high-fat diet, while administration of exogenous IL-22 reversed many metabolic symptoms, including hyperglycaemia and insulin resistance [226]. IL-22 shows diverse metabolic benefits, as it improves insulin sensitivity, preserves the gut mucosal barrier and endocrine functions, decreases endotoxaemia and chronic inflammation, and regulates lipid metabolism in liver and adipose tissues [226]. Similarly, mice treated with an AhR agonist or with *Lactobacillus* strains with high AhR-ligand production capacity exhibited improvement in symptoms of colitis and metabolic syndrome [74, 178]. These treatments alleviate inflammatory insults in mice subjected to experimental colitis [74] and reduce glucose dysmetabolism and liver steatosis in both dietary and genetic animal models of metabolic syndrome [178]. Mechanistically, metabolic improvement is linked to the restoration of intestinal barrier function and the production of the intestinal hormone incretin [178].

The proportion of *Lactobacillus* is also decreased in the microbiota of CRC patients [191, 215], while a number of *Lactobacillus* species exhibited notable anti-carcinogenic effects via the inactivation of microbial enzymes with procarcinogenic activities, such as β-glucuronidase and nitroreductase [227]. To date, no studies have shown that the ability of some *Lactobacillus* species to produce AhR ligands contributes to the anti-carcinogenic potential of these bacteria. However, this mechanism cannot be excluded because AhR is an important node for the development of cancer. Indeed, in comparison to APC^Min/+^ mice that spontaneously developed intestinal tumours, APC^Min/+^/AhR^+/−^ mice exhibited increased tumour incidence, suggesting a tumour suppressor role for AhR. Moreover, a diet rich in AhR ligands can prevent or decrease CRC in mice: first, by inhibiting the Wnt-β-catenin pathway, which is known to be crucial for the proliferation of intestinal stem cells [228], and second, by regulating components of the DNA damage response (DDR) in epithelial stem cells through a mechanism that involves IL-22 production by intestinal immune cells [229]. DDR culminates in either transient cell-cycle arrest and DNA repair or elimination of damaged cells by apoptosis, thereby inhibiting the development of mutations that can lead to CRC [229].

Collectively, these data strongly support the AhR/IL-22 axis a key regulator of intestinal homeostasis. Hence, whether an NP-induced imbalance in this signalling pathway along the microbiota-immune axis could be a first cause of disease development and/or maintenance needs to be further examined in future studies. As noted above, because an NP-induced depletion in AhR-producing bacterial strains is commonly reported (Tables 1 and 5), one may hypothesize that this change represents the missing mechanistic link for colon cancer development in rodents after long-term treatment with a food-grade form of TiO_2_ (i.e., the food additive E171). Cancer evolves through a sequential process from normal cells to preneoplastic lesions before tumour development. Among rats exposed for 100 days to a commercial E171 vial (≈45% of TiO_2_-NPs by number) at a relevant dose for humans, nearly 40% of the animals developed spontaneously preneoplastic lesions in the colon (i.e., initiation of premalignant colorectal lesions), and a steady, low level of inflammation was reported in the colon mucosa [116]. Furthermore, the same study showed increased severity of preneoplastic lesions in E171-treated rats pretreated with dimethylhydrazine to initiate colon carcinogenesis, suggesting the promotion of disease development by TiO_2_ [116]. This effect was confirmed in mice by using a similar E171 batch and showing exacerbated tumour formation (i.e., colorectal adenoma) in a chemical colitis-associated cancer model [230]. These TiO_2_ initiator and promoter effects of preneoplastic lesions in the colon could be due to intestinal micro-inflammation [116] and defective expression of genes involved in oxidative stress, immune response and cancer [231, 232] as well as due to alteration of the intestinal microbiota, as reviewed herein.

In the context of dysbiosis as a susceptibility factor for CRC development, a review of human data clearly shows a reduction in the levels of SCFA-producing bacteria in the microbiota of patients, namely, *Faecalibacterium*, *Roseburia* and *Bifidobacterium,* as well as *Lactobacillus* (Table 5). Decreased abundances of these specific strains was observed in IBD and obese patients, while similar depletion appears in rodents orally exposed to TiO_2_, Ag, SiO_2_ and ZnO NPs, as indicated in Tables 1 and 5. The SCFAs, mainly acetate, propionate and butyrate, are absorbed in the intestine. First, butyrate serves as a major source of energy for colonocytes and for epithelial renewal [233–235], whereas propionate and acetate reach the liver via portal circulation. Propionate is primarily used in gluconeogenesis [233, 234, 236], while in the case of acetate, this organic product enters systemic circulation to reach peripheral tissues, where it serves as a substrate for cholesterol synthesis [233, 236]. As previously detailed, SCFAs exhibit anti-inflammatory effects but are also involved in different physiological processes. Acetate participates in the de novo synthesis of lipids in colonic epithelial cells [237], and similar to propionate, butyrate reduces food intake and stimulates the formation of the anorexigenic hormone leptin [234, 236, 238]. Butyrate has been shown to enhance intestinal barrier function and regulate cellular apoptosis, cell proliferation and differentiation [239]. Moreover, in mice, treatment with butyrate improves insulin sensitivity and increases energy expenditure, leading to reduced obesity [240]. In addition to these anti-obesogenic properties, both butyrate and propionate play a protective role against IBD [241–244] and colon carcinogenesis [245–248]. Altogether, these observations suggest that NP-related depletion of bacteria responsible for SCFA production could be viewed as an additional risk factor for the development of these diseases.

Finally, it should be noted that the inflammatory state induced by the absence of a healthy butyrate-producing microbiota leads to increased expression of the gene encoding nitric oxide synthase, *Nos2*, as well as of nitrate production by the host, a substrate favouring the growth of Enterobacteriaceae belonging to the Proteobacteria [249]. The proportion of Proteobacteria is increased in the gut microbiota of patients suffering from IBD, obesity or CRC (Table 5), and some authors have proposed that the abundance of Proteobacteria as a “microbial signature” of disease progression [182]. The role of Proteobacteria in inflammation has been revealed in various mouse models of obesity, colitis and colitis-associated colorectal cancer. For example, the resistance of GF mice to the development of an obesity phenotype after being fed a high-fat diet was overcome by inoculation of these mice with an *Enterobacter* population isolated from the obese human gut, highlighting the obesogenic potential of Proteobacteria [250]. In mice that exhibit spontaneous development of colitis, such as those lacking Toll-like receptors-5 (*Tlr5*^−/−^), IL-10 (*Il-10*^−/−^) or the transcription factor T-bet and the recombinase activating gene Rag (*T-bet*^−/−^ x *Rag2*^−/−^), an increased intestinal level of Proteobacteria was reported [251–255]. Inoculation of WT mice with two *Enterobacteriaceae* species isolated from faeces of *T-bet*^−/−^ x *Rag2*^−/−^ mice induced intestinal inflammation leading to colitis [253]. In addition, monocolonization with the commensal *E. coli* NC101 of GF *Il-10*^−/−^ mice treated with the colon-specific carcinogen azoxymethane promoted the development of invasive carcinoma [256, 257]. These data indicate that some Proteobacteria are able to induce intestinal inflammation that may promote IBD and/or CRC development. Notably, in recent studies, microbiota composition analysis concluded on a Proteobacteria bloom in mice orally exposed to inorganic NPs (Tables 1 and 3). However, the shift towards pathogenic bacterial colonization is not specific to NP exposure because such a shift was also observed with other xenobiotics. For example, mice exposed to artificial sweeteners and emulsifiers added to many processed foods or to chlorpyrifos (an organophosphorus pesticide frequently detected in the diet) exhibit a high abundance of Proteobacteria, a scenario that predisposes mice to obesity [258–260], colitis [259, 261] and colon cancer [262]. Despite in vivo studies demonstrating that oral exposure to NP induces inflammatory [107, 116, 155, 161, 230] or metabolic [263–265] effects which could be related to IBD, CRC and obesity, it is not known whether the NP-induced bloom of the pathogenic phylum Proteobacteria may also promote the development of one or more of these diseases. In such a context, it should also be investigated whether NP-related GALT dysfunctions could be a cause or consequence of intestinal dysbiosis and whether specific NPs, alone or in mixture, are associated with different disease phenotypes, rendering the host highly vulnerable to IBD, CRC or metabolic disorders (Fig. 2).

## Conclusions

The increasing use of NPs in the food chain, as additives or incorporated into food packaging, has led to concerns regarding the daily exposure of consumers. On the basis of reports presented in this review, one may suggest that the antimicrobial and/or immunotoxic properties of inorganic NPs have the potential to alter the intestinal microbiota and GALT functions, interactions of which are important for many physiological processes in the organism (Fig. 2). This review has summarized the impacts on the gut microbiota-immune axis of the four most common NPs found in the food sector. The existing data highlight a recurrent microbiota signature for nano-Ag, TiO_2_, ZnO and SiO_2_, characterized by an alteration of the F/B ratio, a depletion of *Lactobacillus* strains (SCFA and AhR ligand producers) and an increase in the abundance of Proteobacteria, which may resemble the microbiome shift in IBD, CRC or obesity where gut dysbiosis play a key pathogenic role. These observations raise the need for additional studies for the re-evaluation of NP-containing food additives used for decades in foodstuffs, especially given the uncertainties associated with the long-term effects of these NPs on the gut microbiome as described in this review. In Europe, the new guidance document by EFSA on risk assessment for the application of nanotechnologies in food and the feed chain highlights the need for studies on the composition of the microbiome for nanomaterials, especially for those with antimicrobial properties [266]. This caution appears to be of particular importance given the limited absorption of insoluble particles from the intestine (e.g., TiO_2_, SiO_2_), meaning that the non-absorbed fraction of NPs remains in direct contact with the resident bacteria before being excreted. In this context and based on available data, the NP-microbiota interactions appeared to be localization dependent along the gut, probably due to differences in microbial composition and density from the small intestine to the colon. Changes in the physicochemical properties of NPs during gut transit (i.e., differences in pH and influence of the food matrices and biliary acids) or the extent of particle dissolution for soluble materials (i.e., Ag, ZnO), could modify their long term impact on the microbiome. Except for few studies, the effects of foodborne NPs on the metabolic activity of the microbiota remain largely unexplored; however, this parameter is crucial for evaluating biological consequences for the host and potential hazards. Moreover, most studies have been conducted using pure nanoparticulate matter as NP models instead of the food additives that often exhibit mixed submicron- and nanosized particles, and at high doses, i.e., far above human dietary levels for the equivalent food-grade forms; these aspects could elicit different effects on the gut microbiome. It also appears that interpretations of findings from animals to humans are very limited due to differences in gut microbiota composition and activity. However, as 85% of the 16S rRNA sequence dataset for the mouse microbiota represents genera that are not present in humans [199], the use of GF mice inoculated with the human microbiota seems to be a good model for investigation of the chronic impact of foodborne NPs on bacteria that colonize the human intestine. Some studies used a custom colon reactor in this way, but the exposure time of the microbiota to the NPs was short [113, 117] and hence was not representative of chronic exposure conditions. Furthermore, available data are often limited to the impact of NP models/food additives exclusively on the intestinal flora, bypassing the importance of the microbiota-immune system axis for the host health that would require more integrated approaches as microbiota transfer in GF mice.

Another area that requires detailed investigation is the ability of the gut microbiota to recover after the end of NP exposure; such an investigation would help determine whether NP-induced dysbiosis is permanent and has long-lasting consequences for the host. Notably, the period of exposure to NPs during life is rarely taken into account, while exposure when the microbiota and immune system begin to interact, such as the perinatal period, could induce higher alteration of microbiota-GALT crosstalk than a similar exposure experienced during adulthood. One of the other limitations of the current studies reviewed is that the intestinal immune response (GALT) and gut microbiota alterations were explored after exposure to one type of NP. However, humans are continually exposed to different foodborne NPs as well as to a multitude of other xenobiotics that may have synergistic or antagonistic effects on these functions. One of the challenges in the coming years will be to evaluate the effects on humans of this complex exposome, taking into account exposure to foodborne mineral particles with different physicochemical characteristics. For effective risk assessment, an understanding of the effects of this exposome extended to inorganic (nano) particles will be essential for prevention and remediation strategies and for facilitating the design of safe nanomaterials devoid of biocidal activity when used as food additives or food supplements.

## Data Availability

Not applicable.

## Abbreviations

Ag: Silver
AhR: Aryl hydrocarbon receptor
AMPs: Antimicrobial peptides
bw: Body weight
Card9: Caspase recruitment domain 9
cfu: Colony forming units
CRC: Colorectal cancer
d: Day
DCs: Dendritic cells
DDR: DNA damage response
EFSA: European Food Safety Authority
F/B: Firmicutes/Bacteroidetes
GALT: Gut-associated lymphoid tissue
GF: Germ-free
GI: Gastrointestinal
GRAS: Generally recognized as safe
IBD: Inflammatory bowel diseases
IECs: Intestinal epithelial cells
IELs: Intraepithelial lymphocytes
IFN-γ: Interferon-gamma
IgA: Immunoglobulin A
IL: Interleukin
ILCs: Innate lymphoid cells
LP: Lamina propria
M cells: Microfold cells
MET-1: Microbial ecosystem therapeutic-1
MLNs: Mesenteric lymph nodes
NPs: Nanoparticles
PPs: Peyer’s patches
Reg3: Regenerating islet-derived 3
ROS: Reactive oxygen species
SCFAs: Short-chain fatty acids
SFB: Segmented filamentous bacteria
Si: Silicon
SiO_2_: Silicon dioxide
Th: T helper
Ti: Titanium
TiO_2_: Titanium dioxide
Tlr5: Toll-like receptor 5
TNF-α: Tumour necrosis factor alpha
Treg: Regulatory T-cell
WT: Wild-type
ZnO: Zinc oxide
